# The Role of Predictive Biomarkers in Modern Prostate Cancer Radiotherapy: A Literature Review on Personalised Treatment Strategies and the Prediction of Adverse Effects

**DOI:** 10.3390/life15071062

**Published:** 2025-07-02

**Authors:** Jelena Stanić, Ivana Šović, Luka Jovanovic, Ivana Z. Matić, Predrag Nikić, Marina Nikitović

**Affiliations:** 1 Department of Radiation Oncology, Institute for Oncology and Radiology of Serbia, Pasterova 14, 11000 Belgrade, Serbia; 2 Faculty of Medicine, University of Belgrade, Dr. Subotića Sr. 8, 11000 Belgrade, Serbia; 3 Department of Radiological Diagnostics, Institute for Oncology and Radiology of Serbia, Pasterova 14, 11000 Belgrade, Serbia; 4 Department of Experimental Oncology, Institute for Oncology and Radiology of Serbia, Pasterova 14, 11000 Belgrade, Serbia; 5 Clinic of Urology, University Clinical Center of Serbia, 11000 Belgrade, Serbia

**Keywords:** prostate cancer, radiotoxicity, radiosensitivity biomarkers, personalised treatment

## Abstract

Prostate cancer is one of the most prevalent malignancies in men, posing a significant public health challenge due to its high incidence and long-term treatment-related toxicities. Long-lived patients often experience prolonged side effects that can severely diminish their quality of life. Despite advancements in radiotherapy techniques like IMRT and VMAT, some patients still experience acute and late side effects. Current treatment protocols do not account for individual variability in normal-tissue radiosensitivity, highlighting the need for predictive tools and a personalised treatment approach. Genetic factors and molecular regulators like microRNAs (miRNAs) contribute to these variations by influencing DNA repair, inflammation, and apoptosis. This review explores potential biomarkers of radiotoxicity, focusing on immune-related factors such as IL-6 and TGF-β1, SNPs influencing radiosensitivity, miRNAs involved in radiation responses, and functional assays including the radiation-induced lymphocyte apoptosis (RILA) test. These approaches offer promising tools for identifying radiosensitive patients and enabling risk-adapted radiotherapy.

## 1. Introduction

Prostate cancer is the second most common malignant tumour, after lung cancer, and the fifth leading cause of cancer death in men worldwide. At some point in their lives, around one in eight men may receive a diagnosis of this cancer. Data suggest that prostate cancer imposes a substantial public health burden due to the significant proportion of older men in the general population. The incidence of prostate cancer is three times greater in developed countries than in developing countries [1,2,3].

In Serbia, there has been a continuous increase in cancer incidence and mortality, with 41,578 new cancer cases and 19,350 new cancer-related deaths in 2022. Prostate cancer was the fifth most common cancer both in terms of incidence and prevalence, and it ranks third in terms of incidence and mortality among Serbian men. This unfavourable epidemiological situation, along with the rising number of new prostate cancer patients, can be attributed to the lack of a national screening programme, limited media efforts to raise awareness among men, and an overall lack of sufficient public information [4]. The differences in prostate cancer incidence versus mortality are largely influenced by the extent of PSA screening, which predominantly detects cancer at a localised stage when treatment outcomes are more favourable [5].

In general, when diagnosed on time, most patients with prostate cancer have a long life expectancy after diagnosis. Furthermore, improved therapeutic alternatives for localised prostate cancer have led to higher cure rates, with various treatment options achieving similar outcomes but different toxicity profiles. As a result, some patients may never experience health issues or die from complications directly related to the progression of their disease. However, many patients do live longer with complications from their treatment, which greatly affects their quality of life. Determining the incidence and severity of the toxicity of each therapeutic modality is critical for making decisions about appropriate treatment. Considering all that is mentioned above, long-term radiation-induced toxicity is emerging as a critical concern that cannot be overlooked [6,7].

## 2. The Modern Radiotherapy of Prostate Cancer

Prostate cancer is best treated by a multidisciplinary team of specialists, consisting of urologists, radiation oncologists, and medical oncologists, who collaborate to provide comprehensive care. The treatment modality is decided based on the stage of the disease, histopathological characteristics of tumours, survival benefits, and possible side effects of each of the therapeutic options. However, regardless of the stage of the disease, a patient’s socioeconomic status, personal preferences, and clinical practice patterns at various medical centres represent important factors in selecting the appropriate therapy [6,8]. Current treatment recommendations for prostate cancer, according to the NCCN guidelines, are summarised in Table 1 and emphasise a risk-adapted approach, ranging from active surveillance in low-risk cases to more-aggressive treatments such as surgery, radiation therapy, and androgen deprivation therapy in intermediate- and high-risk patients [9].

It is important to emphasise that in the era of personalised oncology therapy, the introduction of precise diagnostic technologies, along with the increasing availability of safe and effective localised non- or minimally invasive treatment options, has led to a rise in the incidence of and clinical interest in oligometastatic prostate cancer. The definition of oligometastatic prostate cancer is somewhat inconsistent; it ranges from the presence of a single metastasis to between three and five metastases. However, most prospective studies use the definition of three or fewer metastatic lesions with predefined locations [10]. Moreover, the treatment approach for these patients is increasingly shifting towards a more-aggressive strategy. Numerous retrospective studies have demonstrated that in the metastatic stage of the disease, procedures such as radical prostatectomy and the local radiotherapy of metastases can be performed with minimal risk of toxicity, and may improve therapeutic outcomes.

The management of oligometastatic prostate cancer, characterised by a limited number of metastatic sites, remains challenging, with cytoreductive surgery and radiotherapy being the main treatment options. Recent research suggests that cytoreductive surgery may offer better cancer-specific and overall survival compared to radiotherapy, although progression-free survival appears to be similar between the two. Both treatments have manageable side effects, supporting their use in clinical practice. These findings highlight the importance of personalised treatment decisions and the need for further studies to refine therapy for this patient group [11].

**Figure 1:**
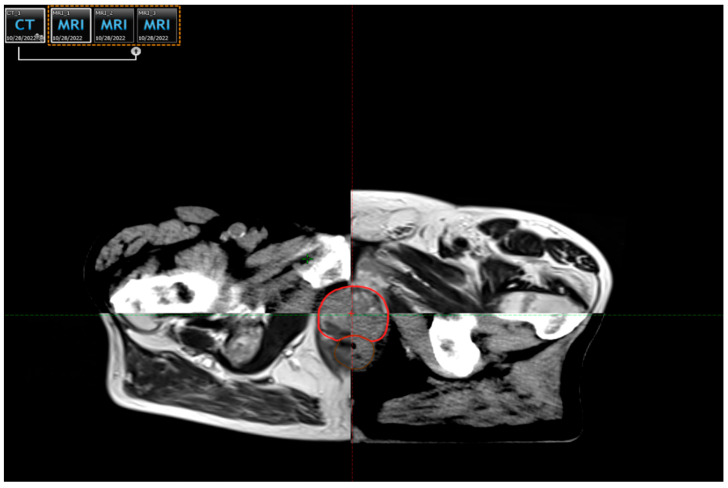
Radical prostate cancer radiotherapy using the VMAT technique: the clinical target volume (CTV), marked in red, was delineated using co-registered magnetic resonance imaging (MRI), ensuring precise mapping and accurate treatment delivery (material from the Institute for Oncology and Radiology of Serbia).

**Figure 2:**
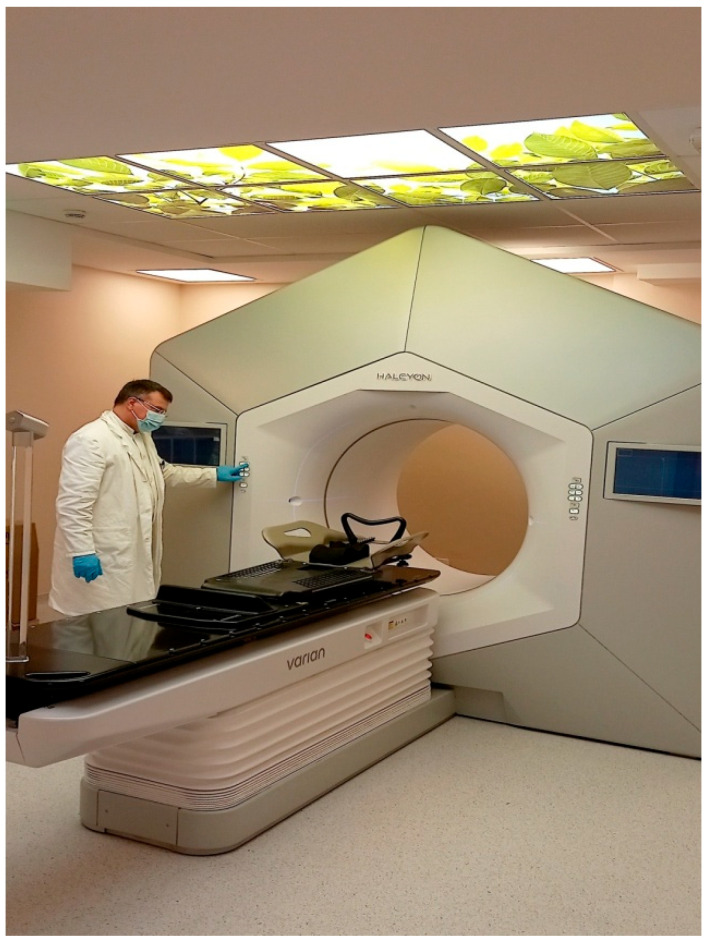
The medical linear accelerator (LINAC) for IMRT and VMAT radiotherapy (material from the Institute for Oncology and Radiology of Serbia).

Radiotherapy, whether used alone or in conjunction with systemic therapy and surgery, plays a crucial role in the management of prostate cancer, reducing the risk of local recurrence and improving overall survival. It is utilised in the treatment of nearly 50% of prostate cancer patients, and approximately 40% of long-term cancer survivors have received radiotherapy at some stage of their treatment journey [12,13]. Depending on the treatment goal, radiation therapy can be classified as radical, postoperative (adjuvant or salvage), or palliative. In terms of its delivery, radiation therapy for prostate cancer can be administered as external beam radiation therapy (EBRT) or as brachytherapy (BT). In radical treatment, radiotherapy is delivered in one, two, or three phases depending on the target volume: prostate only (one phase), prostate and seminal vesicles (two phases), or prostate, seminal vesicles, and regional lymph nodes (three phases). Conventional fractionation (1.8–2 Gy per fraction) is used, with total doses ranging from 72 up to 80 Gy, based on institutional protocols. In postoperative settings, radiotherapy is usually delivered in a single phase with 64–66 Gy to the prostate bed or up to 70 Gy for macroscopic disease. If prophylactic nodal irradiation is indicated, treatment may be delivered in two phases [6,8,9]. External beam radiation therapy is currently one of the most-effective treatments for localised prostate cancer, being used in almost one-third of men with this diagnosis. When planning radiotherapy, the risk of radiation-induced damage to nearby healthy tissues determines the dose limits. The aim is to deliver the highest possible dose to the tumour while minimising exposure to surrounding normal tissue. Modern, highly precise techniques like intensity-modulated radiation therapy (IMRT) and volumetric modulated arc therapy (VMAT) are considered the gold standard for treating patients with prostate cancer. These advanced radiotherapy techniques enable the precise delivery of high radiation doses, up to 80 Gy, while minimising exposure to surrounding healthy tissue (Figure 1 and Figure 2) [14,15,16].

Although surrounding organs at risk, especially the bladder and rectum, are better protected during radiation therapy, some radiation still affects healthy tissue. In prostate cancer radiotherapy, the most clinically significant toxicities are those that lead to treatment interruptions, thereby prolonging the overall course of therapy and adversely affecting patients’ quality of life. Gastrointestinal (GI) toxicity arises from damage to the rectum and bowel, while genitourinary (GU) toxicity results from injury to the urethra, bladder, or prostate. Gastrointestinal symptoms can range from mild issues such as increased bowel frequency to more-severe complications like rectal bleeding, anal pain, or fistula formation. Genitourinary toxicity may present as frequent urination, haematuria, dysuria, urinary incontinence, or urethral stricture [6,7,8].

Consequently, 5–10% of patients may experience severe side effects, resulting in complications such as fibrosis, necrosis, atrophy, and vascular damage. Additionally, among the serious long-term adverse effects of radiotherapy are secondary malignancies. Although secondary cancers are uncommon after radiotherapy for pelvic malignancies such as prostate cancer, they still represent a potentially serious late adverse effect, even in the era of advanced radiotherapy techniques [17,18]. Recent studies on radiation toxicity have identified multiple factors that may influence the risk of complications following radiotherapy. These include the specific radiotherapy technique used, patient age, smoking and alcohol consumption habits, and clinical parameters, such as disease stage, a history of transurethral resection of the prostate, prior abdominal or pelvic surgeries, and the presence of comorbidities [19,20,21,22,23,24,25]. Despite an improved understanding of these contributing factors, individual radiosensitivity remains unpredictable. Current radiotherapy protocols do not account for this variability, highlighting the need for reliable biomarkers to assess individual radiosensitivity prior to treatment [18].

## 3. Individual Radiosensitivity: The Role of Biomarkers and Predictive Assays in Personalising Radiotherapy for Prostate Cancer Patients

In the era of personalised medicine, one of the main challenges in radiation oncology is the discovery of biomarkers that reflect individual sensitivity to radiation. Since it is estimated that 80% of individual differences in radiotherapy toxicity to normal tissues are attributed to a combination of genetic and epigenetic factors, there is an increasing need for a specific test that can accurately reflect individual radiosensitivity and predict the likelihood of radiotherapy-related toxicity [26]. These biomarkers are essential for determining radiosensitivity prior to treatment and enabling individualised risk assessment, which could in turn help optimise treatment planning. For example, it would allow for higher tumour doses in patients with radiation-resistant normal tissue, while those at greater risk of severe radiation toxicity could be considered for alternative approaches. These may include modifying the radiotherapy regimen (e.g., alternative fractionation schemes), switching to a different treatment modality (such as surgery instead of radiotherapy), or introducing pharmacological therapy early to manage symptoms [27,28].

Cellular damage caused by ionising radiation triggers the activation of proteins involved in DNA repair, cell death, inflammation, and other pathophysiologic responses, which contribute to the development of radiotherapy side effects [29]. These effects are classified as acute, subacute, or late: acute effects appear within 1–2 weeks of treatment due to inflammation or the loss of rapidly dividing cells, while late effects, such as fibrosis, vascular injury, or even secondary cancers, may appear months or years later as a result of slower tissue responses and residual DNA damage [28,29].

Potential biomarkers are measurable biological indicators that reflect normal physiological processes, pathological changes, or responses to environmental or therapeutic exposures [29]. In the context of radiotherapy, biomarkers often detected in blood or saliva can reveal molecular events triggered by ionising radiation, such as DNA damage, inflammation, or cell death pathways. These biological factors are valuable for understanding individual radiosensitivity, optimising treatment plans, and minimising toxicity. Predictive assays are functional tests, often performed in vitro, that evaluate how a patient’s cells respond to ionising radiation. These assays commonly assess endpoints such as clonogenic survival, DNA damage and repair capacity, chromosomal aberrations, or radiation-induced apoptosis. Their primary objective is to predict the risk of normal-tissue toxicity and aid in the personalisation of radiotherapy. By assessing the dynamic cellular response to radiation, predictive assays offer direct insight into an individual’s susceptibility to radiation-induced tissue damage [27,28,29].

### 3.1. The Role of Cytokines as Potential Biomarkers for Radiation Toxicity

Cytokines play a central role in the molecular response to ionising radiation, acting as key mediators of inflammation and tissue damage. Inflammatory processes are increasingly recognised as key modulators of malignant tumour responses to radiotherapy [30]. These immunoregulatory proteins, including interferons (IFNs), interleukins (ILs), and growth factors, facilitate communication between cells and can trigger autocrine, paracrine, or endocrine responses [30]. Radiation-induced tissue injury leads to changes in cytokine expression, which closely correlate with both acute and late toxicities through the activation of inflammatory signalling pathways. Circulating cytokine levels therefore reflect the extent of cellular and tissue damage caused by radiation. Because of this, cytokines are increasingly recognised as valuable biomarkers for evaluating normal surrounding tissue reactions to radiotherapy. The interplay between pro- and anti-inflammatory cytokines plays a key role in shaping therapeutic response, influencing the severity of radiation-induced side effects and the potential for tumour resistance [31]. In prostate cancer, in particular, altered cytokine profiles have been linked to radiation-induced toxicity, highlighting their potential for guiding personalised treatment approaches [30,31].

Early studies were the first to highlight the role of cytokines in radiation-induced toxicity, showing in preclinical and clinical lung models that levels of IL-1, transforming growth factor (TGF)-β, and tumour necrosis factor (TNF)-α increased immediately after radiation exposure, while persistently elevated TGF-β was associated with a higher risk of pulmonary fibrosis [32]. Previous studies have reported elevated circulating levels of proinflammatory cytokines, including IFN-γ, IL-6, TNF-α, and IL-4, during the course of radiotherapy [32]. Furthermore, in patients with prostate cancer, radiotherapy has been shown to induce an inflammatory response, characterised by increased serum concentrations of IL-6, IL-8, TNF-α, and TGF-β [33]. The study conducted by Christensen et al. on a cohort of 42 patients with prostate cancer treated with IMRT [30] demonstrated a significant rise in serum levels of IFN-γ and IL-6 during radiotherapy in prostate cancer patients. Additionally, elevated levels of IL-1 and IL-2 were linked to an increased risk of developing genitourinary and gastrointestinal radiotoxicity. Their observations suggest that IL-6, IFN-γ, IL-1, and IL-2 may serve as key cytokine markers associated with radiation responses, particularly in the development of acute gastrointestinal and genitourinary toxicity. It has also been shown that there are differences in cytokine levels between patients with prostate cancer and those with benign prostate lesions or healthy individuals, as well as changes in cytokine levels following radiotherapy and/or androgen therapy [34,35]. These findings provide a foundation for future prospective radiotherapy trials aimed at validating the predictive value of these cytokines, with the potential to inform and personalise patient management during treatment.

Changes in proinflammatory cytokine levels have been linked not only to radiation-induced toxicity but also to the tumour response to radiotherapy. More-aggressive tumour behaviour and treatment resistance have been linked to elevated IL-6 levels. Targeting IL-6 therapeutically may improve treatment results and increase tumour sensitivity to radiation [36]. Patients receiving radiation therapy for many different types of cancers have been found to have a higher proportion of regulatory T cells (Tregs), and this increase is associated with a poor response to treatment [37]. However, the complex role of proinflammatory cytokines in radiation therapy-induced toxicity has not yet been fully understood.

In a study by Stanojkovic et al. [38], the evaluation of potential cytokine signatures in prostate cancer patients revealed significantly elevated circulating levels of IL-6 and IFN-γ in those receiving definitive radiotherapy compared to patients treated postoperatively. This difference may reflect the higher radiation dose used in the definitive group (72 Gy vs. 66 Gy in the postoperative and salvage groups, respectively), variations in irradiated volumes, or factors such as the presence of the prostate and its response to irradiation. In a cohort of 44 patients evaluated at six time points, both univariate and multivariate analyses, adjusted for time point and treatment type, demonstrated a significant association between increased IL-6 levels during radiotherapy and higher grades of acute genitourinary toxicity. The findings also indicate that temporal changes in cytokine levels during treatment, rather than their absolute values, may serve as more-reliable indicators of radiotoxicity.

A recent study on prostate cancer patients undergoing radiotherapy found a significant association between diabetes mellitus and increased acute genitourinary toxicity [39]. In addition, prostate cancer patients who were smokers reported higher maximum fatigue levels than patients who were non-smokers [39]. Interleukin-6 levels rose significantly after the 25th fraction of radiotherapy, and both IL-6 and TGF-β1 levels, measured before radiotherapy and after the 25th radiotherapy fraction, were positively correlated with genitourinary toxicity grades. Additionally, IL-6 and TGF-β1 concentrations after the 25th fraction were associated with fatigue scores [39]. It has also been shown that previous surgical interventions may contribute to the development of radiation toxicity at sites distant from the surgical incision, primarily through the action of TGF-β1 [38]. Although all patients had the same prostate cancer diagnosis, differences in comorbidities and lifestyle factors likely influenced their cytokine profiles. A study by Singh et al. on a cohort of 18 patients with prostate cancer treated with IMRT demonstrated an increase in IL-6 and TNF-α concentrations measured at the end of and 3 months after radiotherapy with an increase in the grade of acute genitourinary and gastrointestinal radiotoxicity, while TGF-β concentrations decreased with an increase in the grade of acute genitourinary and gastrointestinal radiotoxicity [40].

Many studies identified IL-6 as one of the most important cytokines for predicting normal-tissue radiotoxicity effects, not only in prostate cancer but also across various other malignancies treated with radiotherapy. Integrating radiotherapy-related factors, clinical indicators, individual patient features, and circulating cytokine profiles, notably IL-6 and TGF-β1, into machine-learning-based predictive models may be beneficial for anticipating unfavourable normal tissue responses to radiation in prostate cancer patients [38,39,41]. The radiation-induced cytokine profiles are specific for each cancer patient since many individual, biological, clinical, and treatment-related factors may create this pattern [31]. The prospective role of cytokines in the prediction of normal-tissue reactions to radiotherapy for prostate cancer needs to be further investigated on larger homogenous cohorts of patients with prostate cancer, treated with the same type of radiation therapy, with the same radiation dose schedules, dose-volume groups, and clinicopathological features, and using the same criteria for the assessment of acute and late radiation toxicity.

The roles of selected cytokines and their associations with radiotherapy-induced toxicity in prostate cancer patients are summarised in Table 2.

### 3.2. The RILA Assay: A Tool for Stratifying Patients by Radiosensitivity to Anticipate Radiation-Induced Toxicity

Various functional assays can be used to assess the radiosensitivity of patients’ cells, such as lymphocytes or fibroblasts, in order to predict the risk of radiation-induced damage to surrounding healthy tissue. It has been shown that the response of patients’ lymphocytes to radiation determined by functional tests is related to the radiosensitivity of normal tissue [27]. Local radiotherapy has a direct cytotoxic effect on circulating lymphocytes as blood flows through the radiation field. After exposure to ionising radiation, lymphocytes die by apoptosis. However, different subpopulations of lymphocytes, such as CD4^+^ T lymphocytes, CD8^+^ T lymphocytes, B lymphocytes, and NK cells, respond differently to radiation. Studies on lymphocyte depletion in patients consistently indicate that B cells are the most radiosensitive, followed by T cells and NK cells. Among T cells, CD8^+^ T cells exhibit greater radiosensitivity compared to CD4^+^ T cells [42]. Numerous studies have shown that the level of apoptosis induced by the ex vivo irradiation of patients’ peripheral blood lymphocytes before radiotherapy is associated with the risk of radiation-induced damage to normal tissues [43,44]. Specifically, there is a negative correlation between radiation-induced apoptosis of T lymphocytes and the development of late toxicity; patients with lower levels of T cell apoptosis tend to experience more-severe side effects compared to those with higher levels of apoptosis [26,43,44].

A key advancement in this area came in 1995, when researchers in Switzerland, led by Prof. Ozsahin and Prof. Crompton, developed a rapid assay to assess intrinsic radiosensitivity by measuring the radiation-induced apoptosis of CD4^+^ and CD8^+^ T lymphocytes. This assay evaluated the quantity of peripheral blood lymphocytes dying by apoptosis following exposure to ionising radiation at a dose of 8 Gy [43]. Then, in 2005, came the first prospective study using the radiation-induced lymphocyte apoptosis (RILA) assay and involved 399 patients with various cancers (primarily breast, head and neck, genitourinary, and gastrointestinal) who underwent curative radiotherapy [44]. T-lymphocyte apoptosis was assessed before treatment, and patients were followed for acute and late toxicity. After 30 months, no correlation was found between radiation-induced apoptosis in T-lymphocytes and early toxicity or survival. Their findings also showed that CD8^+^ T lymphocytes underwent higher levels of apoptosis compared to CD4^+^ T cells following radiation exposure. Importantly, they identified a strong inverse correlation between the RILA value and the occurrence of late radiation toxicity: patients with higher RILA scores, indicating greater apoptosis, experienced fewer grade 2 and 3 late toxicities, while those with lower apoptosis levels were more likely to suffer from severe side effects. Overall, RILA values > 16% were significantly associated with a very low risk of grade ≥ 2 late toxicity. Conversely, RILA values below 10% were strongly associated with severe late complications. This assay highlighted the potential of T lymphocyte-based tests as predictive tools for individual normal-tissue radiosensitivity [44]. This was further confirmed in the phase II multicenter CO-HO-RT trial, which included 150 breast cancer patients. The high RILA scores were again linked to a lower incidence of grade 2 or higher toxicities [45]. The clinical potential of RILA for the prediction of the risk of radiation-induced breast fibrosis was confirmed in a multicenter French trial involving over 500 breast cancer patients [46].

Foro et al. [47] confirmed a significant correlation between the radiation-induced apoptosis of CD4^+^ T lymphocytes and late genitourinary toxicity in 214 prostate cancer patients treated with radiotherapy. A higher RILA score was notably associated with a lower risk of late toxicity. As a result, research that addressed the issue of the prevalence and frequency of radiation toxicity in patients with prostate cancer discovered that the radiation-induced apoptosis of T cells can be utilised to predict gastrointestinal or genitourinary toxicity. The study conducted on a cohort of 50 prostate cancer patients treated with 3D conformal radiotherapy found that patients with a higher score of late gastrointestinal toxicity had higher levels of early apoptotic as well as late apoptotic and necrotic lymphocytes detected after 24 h [48]. Another study reported lower lymphocyte apoptosis rates in prostate cancer patients who experienced late radiotherapy toxicity when compared with patients without toxicity [49]. However, this study analysed 45 patients.

Thus, the radiation-induced apoptosis of CD4^+^ and CD8^+^ T cells might be exploited to identify radiosensitive individuals prior to initiating radiation therapy. Also, an analysis of acute radiotherapy toxicity confirmed, as expected, that the RILA assay does not predict the majority of acute toxicity endpoints. However, somewhat unexpectedly, the assay did show predictive value for acute breast pain [50]. This finding may provide new insights into the underlying mechanisms by which the RILA assay predicts late radiotherapy toxicity [48,49,50].

Furthermore, a multicenter French study involving 383 prostate cancer patients treated with IMRT revealed valuable findings [51]. Among the patients, 63% received radiotherapy to the prostate, 24% post-prostatectomy, and 13% to the pelvic–prostate region; 54% were also given concurrent hormonal therapy. After a median follow-up of 38 months, late urinary and gastrointestinal toxicities (grades 1–3) occurred in 22.7% and 11.7% of patients, respectively. Importantly, higher RILA scores over 15% were significantly associated with a lower incidence of grade ≥ 2 genitourinary and gastrointestinal radiotoxicities, with a 50% reduction in risk when RILA exceeded 24% [51]. In a study by Mališić and colleagues, the apoptosis levels of CD8^+^ T lymphocytes were determined in 67 patients with localised or locally advanced prostate cancer treated with radiotherapy [52]. This study did not find significant correlations between radiation-induced CD8 T lymphocyte apoptosis levels and the severity of late genitourinary and gastrointestinal radiotoxicity. However, the study identified a correlation between a *TGFB1* variant (TT for C-509T) and the highest mean value for CD8^+^ T lymphocyte apoptosis for late genitourinary and gastrointestinal radiotoxicity (close to reaching significance), and a significant correlation between a *TGFB1* variant (ProPro for Leu10Pro) and the highest mean value for CD8^+^ T lymphocyte apoptosis for gastrointestinal radiotoxicity in prostate cancer patients [52].

Finally, a large multicenter evaluation of the RILA test, conducted as part of the EU-funded REQUITE project, represents a major advancement in the clinical validation of predictive biomarkers for radiation toxicity [53,54]. This international, prospective cohort study was carried out across 26 hospitals in eight countries between April 2014 and March 2017, with a target enrolment of 5300 cancer patients, all of whom were followed for at least two years. The RILA assay itself was performed at three major centres: Leicester (UK), Mannheim (Germany), and Montpellier (France), on a key subgroup of 1319 patients, which included 753 with breast cancer, 506 with prostate cancer, and 60 with lung cancer. Preliminary results, presented at the ESTRO Congress in 2019, strongly confirmed earlier findings from French trials: patients with low RILA scores were at a markedly higher risk of developing radiation-induced breast fibrosis. Significant variations in RILA values were also observed across cancer types and smoking status and in patients with comorbid rheumatoid arthritis, where higher apoptosis levels were found in arthritic individuals. These findings underscore the clinical relevance of the RILA test in identifying patients at risk for radiation-related toxicity. Final results from the full study cohort are still awaited and are expected to provide further insights into the broader applicability of this assay [54].

Although clinical evidence supports the use of the RILA assay, its underlying biological mechanisms remain insufficiently understood. Further research is needed to clarify the role of lymphocyte apoptosis in the development of radiotherapy-related toxicity. The potential clinical value of RILA for predicting individual normal-tissue radiosensitivity has been explored in patients with various malignancies, including breast, prostate, lung, head and neck, and cervical cancers [55]. Differences in RILA scores and threshold values observed across tumour types highlight the need for larger, more homogeneous patient cohorts and the application of standardised protocols to validate these findings for each cancer type [50]. From a clinical standpoint, the utility of RILA as a predictive tool for normal-tissue radiosensitivity is still under evaluation, with results from the ongoing REQUITE project expected to provide further insight in the near future [54]. The RILA assay is based on flow cytometry and uses standardised protocols and reagents [50]. It requires only a few millilitres of peripheral blood, has a relatively low cost (approximately EUR 150 per patient) [55], and can be completed within four days. Additionally, samples can be processed immediately upon arrival in the laboratory, making RILA suitable for integration into routine clinical and laboratory workflows.

### 3.3. Genetic Insights into Radiotherapy-Induced Toxicity

The most detrimental cellular effect of radiation is DNA damage. While most DNA lesions are efficiently repaired, unrepaired damage can lead to cell death through necrosis or apoptosis. In contrast, misrepaired DNA damage may result in genomic instability [27,56].

Studies of genetic syndromes associated with mutations in DNA repair pathway genes, such as ataxia telangiectasia, provided the first insights into human variations in radiosensitivity. In addition to the *ATM* (*ataxia-telangiectasia mutated kinase*) gene, other syndromes have been identified as involving mutations in genes responsible for DNA recognition and repair, which are associated with cellular radiosensitivity, genomic instability, and cancer predisposition [56]. Importantly, other syndromes involving genes like *TP53* (*tumour protein p53*), *RB1* (*retinoblastoma 1*), and components of the *NHEJ* (*non-homologous end joining*) pathway further highlight the role of DNA repair in modulating radiation responses. Li–Fraumeni syndrome (LFS) is a rare inherited cancer predisposition syndrome primarily caused by mutations in the *TP53* gene, a tumour suppressor gene that plays a central role in maintaining genomic stability by regulating the cell cycle and initiating apoptosis in response to DNA damage. *Retinoblastoma 1* is another tumour suppressor gene that encodes the pRb protein, which controls cell cycle progression and prevents uncontrolled cell division; mutations in *RB1* are the primary cause of retinoblastoma, an eye cancer typically occurring in children. Non-homologous end joining genes are a group of genes critical for repairing DNA double-strand breaks, particularly in non-replicating cells, and are essential for maintaining genomic integrity.

Defects in these genes can lead to impaired DNA repair, immunodeficiency, and increased radiosensitivity [57].

Furthermore, experimental data from radiosensitive Chinese hamster ovary cell lines carrying mutations in DNA repair genes like *XRCC2*, *XRCC3*, *XRCC4*, and *RAD51C* support the critical role of these loci in determining individual susceptibility to radiotherapy toxicity. These findings collectively underscore the importance of key DNA repair genes in shaping radiosensitivity and radiotherapy outcomes [58].

Given that in real practice 5–10% of patients experience severe side effects of radiation, this suggests that such severe reactions to radiation cannot be explained only by rare genetic disorders that affect few people in the general population. Therefore, it is believed that more common and subtle genetic variations, like single-nucleotide polymorphisms (SNPs), may influence how sensitive a person’s normal tissue is to radiation [59].

Moreover, a retrospective cohort study involving 124 prostate cancer patients treated with three-dimensional conformal radiotherapy explored the association between SNPs in candidate genes related to DNA damage recognition, repair, and steroid metabolism, and the occurrence of clinical radiation toxicity. Notably, variants in the *LIG4*, *ERCC2*, and *CYP2D6* genes emerged as potential predictive markers for identifying individuals at higher risk of radiotherapy complications [60]. Building on this, the critical role of DNA repair mechanisms in modulating radiation-induced adverse effects was further investigated through a prospective study of 406 patients undergoing intensity-modulated radiotherapy. This study monitored acute toxicity by using established clinical criteria while analysing the constitutive mRNA expression profiles of DNA repair genes to assess their ability to predict radiosensitivity or radioresistance. The findings suggested that the higher baseline expression of these repair genes may confer a protective effect against acute radiation side effects, highlighting their potential as biomarkers for individual radiosensitivity [61].

Genome-wide association studies (GWASs) found a region on chr11q14.3 linked to rectal bleeding [62] and one in *TANC1* (*chr2q24.1*) linked to overall toxicity after radiotherapy in prostate cancer patients. However, due to small sample sizes, no single variant was clearly shown to increase side effect risk. Kerns et al. [63] studied over 1500 prostate cancer patients treated with curative radiotherapy. Two years after treatment, 17.8% experienced rectal bleeding, 15.0% had increased urinary frequency, and 8.1% had reduced urine flow. A recently published meta-analysis identified two SNPs: rs17599026 (*5q31.2*), linked to urinary frequency, and rs7720298 (*5p15.2*), with decreased urine stream, both in genes expressed in tissues affected by pelvic radiotherapy. Schack et al. [13] identified nine SNPs associated with late radiation-induced morbidity in 96 prostate cancer patients treated with definitive radiotherapy.

The Radiogenomic Consortium (RGC) [64] meta-analysis included 5456 patients, with 2759 receiving radiotherapy for breast cancer and 2697 for prostate cancer. Eight toxicity outcomes were analysed: total, acute, late, acute rectal, late rectal, acute dermal, telangiectasia, and fibrosis. The study found a significant association between the *ATM* rs1801516 Asn allele and increased radiation-induced toxicity risk [65]. Cesaretti et al. [66] studied whether genetic changes in the *ATM* gene were linked to rectal bleeding in a dose- and volume-dependent manner. The study involved 180 prostate cancer patients who received either brachytherapy or a combination of EBRT with brachytherapy, with at least 1 year of follow-up. They found that genetic variants in the *ATM* gene were associated with an increased risk of radiation-induced proctitis in patients receiving a full tumour dose. Valdagni et al. [67] investigated why some prostate cancer patients experience late rectal bleeding (LRB) despite good rectal dose-volume histograms (DVHs), while others with poor DVHs do not. The study included 30 patients who received conformal radiotherapy with doses > 70 Gy. A comparison of gene expression revealed that nine genes were significantly downregulated in the low-risk bleeder group compared to the high-risk bleeder and nonbleeder groups, and four genes were upregulated in the high-risk nonbleeder group. The study suggested that these genes could help predict sensitivity and resistance to LRB, warranting further validation in larger datasets. Cintra et al. studied 48 prostate cancer patients and found that two *TP53* polymorphisms were associated with radiation-induced toxicity: one linked to acute skin toxicity and the other to chronic urinary toxicity. No such associations were found for *ATM* or *MDM2* polymorphisms [68].

In conclusion, although numerous SNPs associated with radiosensitivity have been identified through candidate gene studies and GWASs, only a limited number have been consistently validated, highlighting the need for further large-scale, well-designed studies.

### 3.4. MicroRNAs and Their Role in Radiation-Induced Toxicity

MicroRNAs (miRNAs) are small RNA molecules that do not code for proteins but regulate gene expression by blocking the translation of messenger RNA (mRNA). They play key roles in many biological processes, such as cell development, programmed cell death (apoptosis), blood vessel formation, cell growth, and metabolism. Due to their high stability in bodily fluids, miRNAs are excellent candidates for use as biomarkers in diagnosing and predicting the progression of diseases, including cancer. Typically, miRNAs are 21–25 nucleotides long and are encoded within the exons or introns of protein-coding genes. Their “seed” region binds to complementary sequences in the 3′ untranslated region (3′ UTR) of target mRNAs, leading to the repression of translation or mRNA degradation, which disrupts protein production and affects both normal and disease-related processes [69]. Moreover, miRNAs show potential for non-invasive diagnostic applications in several cancers, such as colorectal, prostate, and bladder cancer. However, challenges remain in validating and normalising reference values, as well as accounting for variability related to patient age, ethnicity, and sample storage protocols [70,71,72]. A major difficulty is the high variability in miRNA expression between individuals, influenced by factors like genetics, age, ethnicity, health conditions, medications, and environmental exposures. This variability makes it hard to establish universal diagnostic or prognostic thresholds and reduces reproducibility between studies and patient groups [73].

Another significant issue is the lack of standardisation in pre-analytical and analytical procedures. miRNA quantification can vary greatly depending on the type of biofluid analysed (e.g., serum vs. plasma), the method of RNA extraction, the normalisation strategy applied, and the detection platform used (e.g., qPCR, microarray, or next-generation sequencing). According to Ho et al., inconsistencies at any of these stages can lead to substantial variation in results, limiting cross-study comparability and clinical reliability [73].

In the context of prostate cancer, miRNAs are essential regulators of radiotherapy responses, influencing radiosensitivity, radioresistance, and radiation-induced toxicity. As radiosensitivity modulators, miRNAs can either sensitise prostate cancer cells to radiation or contribute to their resistance [74]. Additionally, miRNAs have been implicated in the development of radiation toxicity, particularly in the genitourinary and gastrointestinal systems, through their ability to modulate inflammatory and immune responses [75]. Profiling miRNA expression is increasingly used in cancer diagnostics to predict treatment outcomes, including responses to radiotherapy and adverse effects such as toxicity [76].

Certain miRNAs enhance the effectiveness of radiotherapy in prostate cancer by promoting apoptosis, impairing DNA repair, or inhibiting oncogenic pathways, thus sensitising tumour cells to radiation [69]. Chromosome loss, epigenetic silencing, and *TP53* deficits all affect *miR-34a*, one of the most therapeutically important tumour-suppressive microRNAs [77]. Its key role in carcinogenesis and clinical relevance are explained by this connection with *TP53*. It is now known that *miR-34a* is a tumour suppressor that increases radiosensitivity by targeting *BCL2* and promoting apoptosis. Additionally, a number of other targets, cell cycle regulators such as *MYC*, *CDK4/6*, and *NOTCH1*, are also noted [78]. Moreover, *miR-34a* targets *AXL*, *MET*, *SIRT1*, *CD44*, and *PDL1*, genes that play crucial roles in resistance to cell death, migration, metastatic spread, and immune evasion. By downregulating androgen receptors, *miR-34a* may also have a significant role in prostate and other androgen-dependent cancers [77]. The downregulation of *miR-34a* is commonly observed in various cancers, including prostate cancer, and its restoration can improve treatment outcomes [79]. There is significant interest in the therapeutic targeting of this *miRNA*, and a recent study by Abdelal et al. presented the first fully modified version of *miR-34a* with outstanding stability, activity, and anti-tumour efficacy in vitro [77].

Moreover, *miR-145* inhibits cell proliferation and the epithelial-to-mesenchymal transition (EMT), leading to an enhanced response to radiation. It plays a crucial role as a tumour suppressor, and its downregulation is associated with poor prognoses [80]. *miR-205* enhances radiosensitivity by targeting *PRKCE* (PKCε) and *ZEB1*, which are key regulators of DNA repair and survival signalling [81], whereas *miR-541-3p* increases the DNA damage response and apoptosis after radiotherapy by targeting *HSP27* [82]. Furthermore, *miR-29b-3p* suppresses the *Wnt/β-catenin* signalling pathway, which is crucial for tumour growth and radiation resistance [83].

On the other hand, certain miRNAs are implicated in promoting radioresistance by enhancing DNA repair mechanisms or protecting tumour cells from radiation-induced damage. *miR-21* is among the most abundant and highly conserved microRNAs, with both up- and downregulation observed in several cancers, as well as in cardiovascular and immunological diseases [84]. Although its role as a diagnostic biomarker is limited, it is well established that *miR-21* is upregulated in cancer [85]. The main proto-oncogenic mechanism of *miR-21* is through targeting *PTEN* and activating the *PI3K/AKT* signalling pathway. The *PTEN* gene functions as a tumour suppressor by negatively regulating *PI3K/AKT* signalling, which is critically involved in cellular growth, proliferation, and survival [85]. The loss or inactivation of *PTEN* leads to the dysregulation of this pathway, significantly contributing to the development and progression of various malignancies. Notably, *PI3K/AKT* signalling is one of the most frequently overactivated intracellular cascades in human cancers, underscoring its central role in oncogenesis. Through its downstream effectors, this pathway promotes tumour initiation, progression, invasion, and metastasis, making it a prominent target in cancer research and therapeutic intervention [85]. The upregulation of *miR-21* suppresses programmed cell death and contributes to radioresistance, metastasis, and the development of castration-resistant prostate cancer (CRPC) [86]. Similarly, *miR-95* has been correlated with poor prognoses and radioresistance, facilitating aggressive cancer phenotypes [87]. *miR-106b* promotes cell cycle progression and DNA repair, leading to enhanced resistance to radiation [88]. Furthermore, *miR-210*, *miR-21*, and others are upregulated in response to reactive oxygen species, supporting tumour adaptation and survival in response to radiotherapy [88].

miRNAs are also associated with the development of genitourinary and gastrointestinal toxicities, which are common adverse effects of prostate cancer radiotherapy. Several miRNAs, namely *miR-132-5p*, *miR-197-3p*, *miR-151a-5p*, and *miR-125b*, have altered circulating levels in patients experiencing acute and late toxicities following radiotherapy, indicating their potential as biomarkers for radiation-induced damage [89].

**Figure 3:**
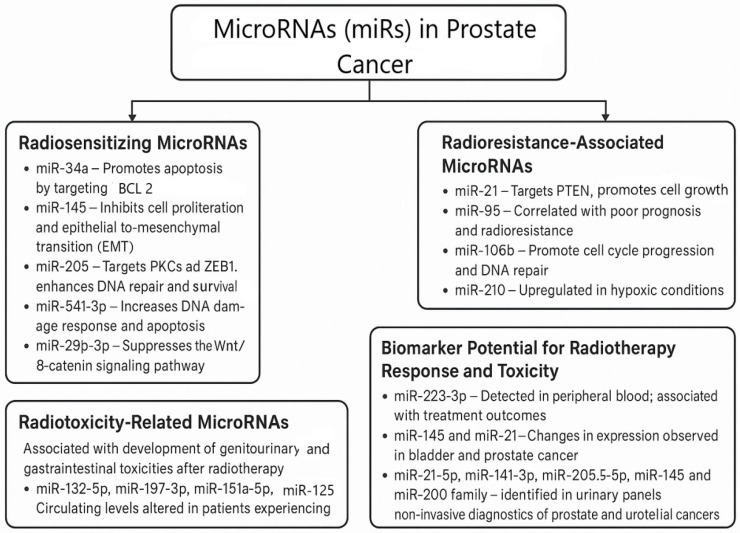
MicroRNAs in prostate cancer: key players and its roles.

Several miRNAs have emerged as promising non-invasive biomarkers for monitoring treatment response and predicting radiation-induced toxicity. Notably, *miR-223*, *miR-145*, and *miR-21*, detected in peripheral blood, have been linked to treatment outcomes across multiple cancer types, highlighting their potential as predictors of radiotherapy efficacy [90]. Additionally, increased levels of urinary miRNAs hold considerable promise for diagnostic screening. A recent systematic review emphasised the significance of *miR-21-5p*, *miR-141-3p*, *miR-205-5p*, *miR-145*, and the *miR-200* family within comprehensive urinary panels, which could serve as effective non-invasive tools for the diagnosis of both prostate and urothelial cancers (Figure 3) [72].

Interestingly, recent findings underscore the complexity of gene function in cancer biology, revealing that the same gene may exert vastly different roles depending on the tumour type, cellular context, and molecular environment. A striking example is NEIL3, a gene implicated in both DNA repair and non-coding RNA regulation. In pancreatic ductal adenocarcinoma (PDAC), its circular RNA isoform, *circNEIL3*, acts as an oncogenic driver by sponging the tumour-suppressive *miR-432-5p*, thereby promoting the *ADAR1*-mediated editing of *GLI1* and facilitating tumour proliferation and metastasis [91]. Conversely, in prostate cancer, the loss of the NEIL3 protein leads to the enhanced activation of the *ATR/CHK1* pathway, which strengthens DNA repair mechanisms and contributes to radiotherapy resistance [92]. This dual behaviour exemplifies how molecular targets such as NEIL3 and associated *miRNAs* may function as either oncogenes or tumour suppressors in a tissue-specific and isoform-dependent manner. Such context-dependent roles highlight the importance of precision oncology and the need for tailored therapeutic strategies that account for the molecular landscape of individual cancers.

## 4. Discussion

The growing interest in personalising radiotherapy necessitates robust, multifactorial tools to accurately predict individual susceptibility to treatment-related toxicity. While single biomarkers such as proinflammatory cytokines (e.g., IL-6, TGF-β1), radiation-induced lymphocyte apoptosis (RILA), or specific single-nucleotide polymorphisms (SNPs) have individually shown promise, their predictive power remains limited when evaluated in isolation. Therefore, combining biomarkers that reflect different biological processes may provide a more comprehensive assessment of individual radiosensitivity, reflecting different biological factors and pathways.

We propose a conceptual framework that integrates these complementary biomarker classes. At the baseline, genomic profiling (e.g., SNPs in DNA damage repair or inflammatory pathway genes such as *TP53*, *ATM*, and *XRCC1*) can provide static information on individual predispositions to toxicity. Concurrently, functional assays like RILA quantify individual radiosensitivity by measuring the apoptotic response of peripheral lymphocytes to ex vivo radiation. During treatment, monitoring dynamic biomarkers such as serum cytokines (e.g., IL-6, TGF-β1) offers insight into tissue inflammation and early systemic responses to radiation exposure.

This integrative approach enables a stepwise risk stratification model in which patients are categorised into low-, intermediate-, or high-risk groups for developing radiotoxicity. Such stratification may ultimately support individualised radiotherapy planning, whether through dose modification, enhanced toxicity surveillance, or early intervention strategies.

The added value of this framework lies in the biological complementarity of its components: germline genetic variants (SNPs), intrinsic cellular radiosensitivity (RILA), and treatment-induced inflammation (cytokines). Future studies should focus on prospectively validating such integrative models and incorporating advanced predictive models (e.g., machine learning) to refine prediction accuracy. Establishing standardised thresholds and harmonising assay methodologies across centres will also be crucial for successful clinical translation in addition to reporting unique radiotoxicity effects.

Each of the proposed potential biological predictors of normal-tissue radiosensitivity could be rapidly evaluated using blood samples: patient-derived lymphocytes or serum and plasma, as a starting biological sample. SNP genotyping, flow cytometry-based RILA, and the determination of cytokine levels by commercially available and reliable ELISA tests are low-cost, cost-effective, and can be easily incorporated into clinical settings after robust research studies and clinical validation.

## 5. Conclusions

Advances in the treatment of localised prostate cancer have improved survival, shifting the focus to minimising long-term side effects that can significantly impact quality of life. Despite the use of modern radiotherapy techniques, some patients continue to experience severe toxicities, highlighting the need for predictive biomarkers to guide personalised care. Current protocols do not take into account individual differences in radiosensitivity. Indeed, a primary goal of translational research in radiotherapy is the identification and validation of biomarkers that can be used to assess the risk of acute and late toxicities, as well as to predict an individual’s sensitivity to radiation. Integrating individual, clinical, and biological characteristics that are unique to each patient into treatment planning ultimately allows clinicians to tailor radiotherapy regimens, thereby balancing the risks and benefits for each patient.

The RILA assay has emerged as a valuable, non-invasive tool for predicting individual radiosensitivity. When integrated into clinical models along with other patient-specific factors, RILA helps tailor treatment strategies that support dose escalation in patients at low risk of toxicity and guide alternative approaches for those at higher risk. Cytokines, such as IL-6, TNF-α, and TGF-β, are also gaining attention as biomarkers of inflammation and radiation-induced tissue damage. Together, these biomarkers offer promising avenues for optimising treatment plans, reducing complications, and improving outcomes. Genetic variations, such as SNPs, likely play a significant role in individual differences in normal-tissue sensitivity to radiation, emphasising the need for personalised approaches in radiotherapy. MicroRNAs significantly influence prostate cancer radiotherapy by modulating radiosensitivity, resistance, and toxicity. Their stability and specific expression patterns make them valuable non-invasive biomarkers for predicting treatment outcomes and side effects. Ongoing research will further define their role in advancing personalised radiotherapy.

Developing machine learning models to predict both acute and late normal-tissue reactions to radiotherapy in patients with various cancers is a vital step toward personalised radiotherapy. Such predictive models should integrate multiple biological factors representing different mechanisms of radiosensitivity: individual patient characteristics, clinical and treatment-related factors, RILA assay results, and a combination of potential biomarkers, such as SNPs in specific genes, gene or miRNA expression profiles, and circulating cytokine patterns. By combining these data, patients can be stratified into subgroups with varying risks of adverse effects, allowing radiotherapy to be tailored and optimised for each subgroup.

## Figures and Tables

**Table 1:** Prostate cancer treatment recommendations based on risk group (adapted from NCCN Guidelines, Version 1.2025) [9].

Risk Group	TNM Stage	PSA (ng/mL)	Gleason Score/Grade Group	Treatment Options
Very low	T1c	<10	GS ≤ 6 (Grade Group 1), <3 positive biopsy cores, PSA density < 0.15	- Active surveillance (preferred) - Radical prostatectomy (selected cases) - Brachytherapy (rare)
Low	T1-T2a	<10	GS ≤ 6 (Grade Group 1)	- Active surveillance (preferred) - Radical prostatectomy - EBRT or brachytherapy
Favourable intermediate	T1-T2b	10–20	GS 3 + 4 (Grade Group 2) OR <50% biopsy cores positive	- Radical prostatectomy ± lymph node dissection - EBRT ± short-term ADT (4–6 mo) - Brachytherapy ± EBRT
Unfavourable intermediate	T2c or ≥50% cores + or GS 4 + 3 (Grade Group 3) or PSA 10–20	10–20	GS 4 + 3 (Grade Group 3) or multiple intermediate factors	- Radical prostatectomy ± pelvic LN dissection - EBRT + short-term ADT - EBRT + brachytherapy boost
High	T3a or PSA > 20 or GS 8 (Grade Group 4)	>20	GS 8 (Grade Group 4)	- EBRT + long-term ADT (2–3 years) - EBRT + brachytherapy + ADT - Radical prostatectomy (select cases, as part of multimodal treatment)
Very high	T3b-T4 or GS 9–10 (Grade Group 5) or >4 biopsy cores with Grade Group 4 or 5	Any	GS 9–10 (Grade Group 5)	- EBRT + long-term ADT ± abiraterone - EBRT + brachytherapy + ADT - Radical prostatectomy (select cases)
Regional (N1)	Any T, N1, M0	Any	Any	- EBRT + long-term ADT - ADT alone (non-curative setting) - Consider abiraterone + ADT
Metastatic (M1)	Any T, any N, M1	Any	Any	- ADT + novel hormonal therapy (abiraterone, enzalutamide, and apalutamide) - ±Docetaxel (in high-volume disease) - Bone-protecting agents (zoledronic acid or denosumab) - Palliative radiotherapy-Lu-177 PSMA, PARP inhibitors (selected mCRPC)

GS: Gleason score; EBRT: external beam radiation therapy; ADT: androgen deprivation therapy; LN: lymph node; and mCRPC: metastatic castration-resistant prostate cancer.

**Table 2:** Summary of key cytokines involved in radiotherapy-induced toxicity in prostate cancer.

Cytokine	Role	Association with Adverse Radiotherapy Effects
TGF-β1 (Transforming Growth Factor Beta 1)	Modulates immune responses and fibrosis	Elevated TGF-β1 levels after radiotherapy can contribute to fibrosis and toxicity in prostate cancer patients [33,39]
TNF-α (Tumour Necrosis Factor Alpha)	Proinflammatory cytokine, regulates apoptosis	High TNF-α levels after radiotherapy may be linked to increased inflammation and risk of late toxicities [33]
IL-6 (Interleukin-6)	Mediates inflammatory responses and tissue repair	Elevated IL-6 can be a marker of poor prognoses, contributing to inflammation and fibrosis post-RT [30,36]
IL-1β (Interleukin-1 Beta)	Promotes inflammation and tissue damage	Associated with radiation-induced fibrosis and increased late toxicities in prostate cancer radiotherapy [39]
IFN-γ (Interferon Gamma)	Enhances immune response and regulates apoptosis	Higher IFN-γ levels may correlate with increased lymphocyte apoptosis and lower late toxicity risk [30,38]
IL-2 (Interleukin-2)	Stimulates T-cell activation and proliferation	Plays a key role in immune recovery after radiotherapy; altered levels may influence long-term toxicities [30]
IL-8 (Interleukin-8)	Chemotactic cytokine that attracts neutrophils	Elevated IL-8 levels after radiotherapy may contribute to inflammation and exacerbate toxicities [33]

## Data Availability

All data generated as part of this study are included in the article.
